# On the Cure of Tooth-Ache, and a Method of Treating Exposed Nerve

**Published:** 1856-01

**Authors:** Donaldson Mackenzie

**Affiliations:** Surgeon Dentist, 21A, Saville-row, Regent-st.


The following article, which we take from the *Dental Recorder* of July, reminds us much of by-gone practice—fifteen or twenty years since, when much attention was first turned to the preservation of teeth with exposed pulp. Capping the nerve was probably then practiced more than at present, and lead was thought to be a much better cap than gold; that as leaden bullets often remained in the body for years without any serious injury, so would it also remain in contact with the pulp without inducing inflammation. This notion is not now, we believe, so generally entertained, and to avoid the use of two metals in the same cavity gold is generally preferred.—But Mr. MACKENZIE appears to prefer the use of Amalgum for filling such teeth.

The article will, however, give our readers some idea of Practice among our English Dentists.

From the London Medical Times and Gazette.

### ON THE CURE OF TOOTHACHE, AND A METHOD OF TREATING EXPOSED NERVE.

BY DONALDSON MACKENZIE, ESQ., SURGEON-DENTIST.

The painful affection called toothache is so well known, that any description would be superfluous. It may, however, not be out of place to distinguish toothache as that pain experienced when the ganglion of vessels contained in the cavitas pulpæ of the tooth is inflamed; and that all other affections arising from the teeth are entirely distinct from this, and require a very different treatment. It would be most convenient were they distinguished by different terms, as—toothache (*)

* Periostosis, or inflammation of dental periosteum, and alveolus in a future paper.

and socket-ache, a species of terminology, although not correctly anatomical, yet sufficiently significant for general use.

True toothache is, therefore, the expression of inflammation acting upon the pulp and lining membrane of the centre of the tooth, and is always produced upon caries approaching that cavity; and, as inflammation of other tissue is reducible by medical interference, it would seem absurd to suppose that the same action upon so minute a structure as the dental pulp, should defy our skill. However, I hope that, with few exceptions, this notion has gone to the tomb of all the Capulets. I am individually of the opinion that the guillotine is as legitimate an instrument for the cure of headache as the forceps for the relief of toothache. When caries has extended to that point at which inflammation of the pulp begins, the presumption is, that this action is set up by atmospheric pressure, or the permeation of irritants through the tubulous structure of the bone to the pulp cavity. This circumstance is sufficient of itself to direct us in curative proceedings; for, if an irritating fluid can find a passage, what is to stop the progress of menstrua combined with principles of an opposite character?

The symptoms expressed by inflammation acting upon any part of our body, which is obvious to our senses, are pain, heat, redness, and swelling; but in the dentinal pulp, as in other internal organs, the increase of heat is not observable, and the acute pain may be the result of this tissue, by swelling, being forced against the unyielding walls of its bony chamber; for we find, upon the pulp being allowed to enlarge, from the destruction of a portion of that chamber, that the pain is very much lessened or entirely removed.

In inflammatory affections of the body, the Medical practitioner in his remedial treatment has recourse to bleeding, purging, blistering, heat, cold, etc., according to the structure of the part affected; but our unfortunate teeth are not often so rationally dealt with, as may be gathered from the fact of some of our first men recommending muriatic acid, or argento-nitrate, as a cure for inflammation (by their own account) in one of the most highly organized structures of the human body. One would be almost led to conceive the pulp to be a fungus to be destroyed, instead of a membrane to be preserved vital with all the skill we possess.

Toothache is dependent on the same causes as general phlegmasia, and may be reduced by the usual treatment of lowering the circulation, but the seat of the disease being confined to so small a spot, it is much more under control, and it will generally yield to local applications of a soothing nature.

All preparations of camphor, opium, morphia, etc., will reduce the violence of toothache ; but success is not always to be relied upon from the use of any one of these singly, frequent applications are sometimes necessary, and also a combination of two or more of them. The *modus operandi* is simply to saturate a suitable piece of lint, and insert it into the cavity ; often one application is sufficient, sometimes a dozen are necessary, but perseverance will ultimately command success.

When a patient presents himself suffering from pain in his teeth, which he of course calls toothache, it is the business of the dentist to examine and determine whether toothache is present, or inflammation of the periosteum; this he may do from an optical survey combined with the expressed symptoms of the patient.

Should the former only be present, the remedy is at hand ; but it may be found that the sockets of one or more of the other teeth are affected, in which case should the pain be severe, (*) the forceps is generally our only resource, and the anodynes for those with inflamed pulp. When pain has ceased in them, the carious portion should be carefully and entirely cut away; the tooth may then, or in a few days subsequently, be stopped in the usual way with gold or amalgam, as the operator may deem most fitting, for the choice of material cannot be left to the patient, as a cavity may be quite suitable to receive the soft stopping, but not able to bear the pressure necessary to solidify gold, from the circumstance of there not being sufficient thickness of unsoftened ivory left to serve as a ceiling to the pulp cavity, or floor of the stopping.

In my paper published in this Journal (October 28th) I observed that caries is generally found to exist between the front teeth when closely crowded together, and where that does exist, both teeth are in most cases more or less affected ; so without making a considerable opening with a file, the difficulty of stopping either is apparent; it is not possible to devise any specific mode in absence of an individual case, so that the skill and judgment of the operator are particularly called into play.

However, we are not frequently left in this dilemma, for it

* Sometimes local abstraction of the blood, or the application of warm water and tincture of opium may give relief, but in most cases the pain returns.

is rare to find two opposite cavities, each equally suitable for stopping. In most cases, before the dentist is called upon, the disease has destroyed one of the teeth sufficiently to enable the operator to stop the other. When this is not the case it is then of course necessary to make an opening, and the features then displayed will determine the mode of treatment. We may suggest, for example, to file the most decayed in the manner described in a former paper, and thus derive space enough to successfully stop the other.

I need hardly add that the care required to perform this operation always correspon dswith the difficulty of its performance. It is needless to discuss the value of the different stoppings here, as for the front teeth there can be no choice. Gold is the only substance that will not communicate a dark shade, or to speak more emphatically, turn the tooth black. This obligation to use gold in such awkward positions calls for an instrument better adapted to intricate work than those usually made use of. When in any such difficulty I generally construct an instrument to suit the case in hand, and the marginal drawing will show an instrument I use for the purpose of stopping with gold cavities situated on the sides betwixt the front teeth, and sometimes the bicuspidati.*

By this drawing it will be seen that the instrument is similar to a pair of forceps having movable bills, one of which is formed to rest against the sound side of the tooth, while the other is the stopping-tool to force in the gold. The advantage it possesses over those in use is, that the force (required to consolidate and fix securely the gold in the cavity) is exerted between the manibles of the instrument. In the ordinary way the force is exerted against the side of the tooth, frequently causing considerable inflammation of the socket, and probable loss of vitality to the tooth itself.

Improvements might be made in this instrument; for instance, if two were made, a right and a left, only one of the bills might be movable, viz: the stopping point, for various sized cavities, etc.

The instrument from which the drawing was made, cannot be without imperfections, as it was formed upon the spur of the moment to suit a case, from a pair of damaged excising forceps.

The basis of all soft stoppings (of any use) is quicksilver

* The instrument, as illustrated by the drawing, is identical with J. D. Chevalier's plugging forceps.—Ed. Rec.

combined with any metal that will concrete after amalgamation. Gold and platina, which are the only metals that do not oxidize, do not concrete after amalgamation; but most other metals do, and in whatever form, become discolored when acted upon by the fluids of the mouth; it is therefore obvious that no metal except gold can be used as a stopping for the front teeth, platina being deficient in pliability; indeed, none of the amalgams now in use should be employed to stop front teeth, and yet I have been at different times called upon to remove them from the teeth of young persons on account of discoloration. Although I object to the use of soft stoppings for the front teeth, and although they were much written against twenty years ago, yet experience teaches us to hail them as a great auxiliary when judiciously applied.

There need not be a question that pure gold, either sponge or leaf, is the true and legitimate succedaneum for decayed cavities, yet many cases are presented to us where an attempt to introduce gold would be attended with great uneasiness to the patient, and probably the subsequent oss of the tooth. For example: in cases where the caries has left the ivory so thin over the cavitas pulpæ, that any attempt to press gold into the carious cavity would cause severe pain by forcing up the roof of the chamber upon the pulp. In cases of this nature it is evident that recourse must be had to a stopping that may be introduced in a plastic state to become subsequently concrete.

A curative process is set up by nature in teeth that have been stopped with the silver amalgam, which, therefore, recommends itself as the most suitable for extreme cases; the only objection that it admits of is its aptitude to discolor the tooth. The "white stoppings" are less objectionable in that respect, but, as they have not been so long in use, their curative virtues have not been so well ascertained.

When the disease has proceeded a stage further, the consequence is, that in cutting away the softened bone, we may expose some portion of the pulp, which at once precludes the possibility of stopping the tooth by any of the ordinary methods.

Mr. Koecker devised a method of cauterizing the exposed pulp, and protecting it by means of a leaden floor. Mr. Snell improved upon the cauterizing instrument, but both plans seem to have fallen short of anticipation, and a sufficient cauterization could not be effected; probably from a combination of unavoidable circumstances—the first, the impossibility of raising the iron to a white heat at a lamp or candle; the second, the impossibility of placing the instrument upon the part, before it had parted with a large portion of the low degree of redness it acquired; the third, the many attempts required from the terror of the patient causing him to withdraw his head in the fear of having his mouth burnt.

It is obviously of consequence to secure the services of any tooth, and some extra trouble taken with a tooth in this state is often satisfactorily bestowed.

That this mode of treating exposed nerve has failed in general success is evident from the circumstance of its disuse by the Profession; but if those gentlemen, Messrs. Koecker and Snell, have succeeded in one case, with the inadequate means then at their disposal, what may we not hope for now, with our resources? I quite agree with Mr. Bell and other writers of eminence, that, could the cauterizing wire be got to the part at a sufficiently high temperature, the operation would be successful. To produce this desirable effect, we have the voltaic pile, and for those who would practise this mode, let them procure a fine piece of platina wire, twist the centre of it into a small flat coil, leaving the two ends free to be attached to the poles of the battery; place in the cells an active solution of acid; have the cavity well freed from moisture; place the platina coil in immediate proximity to the spot intended to be cauterized; let an assistant lower the plates into the trough; when the platina coil is excited to a white heat, touch the protruding pulp steadily, and the desired result will be attained.*

Having found, in the early years of my practice, the method then followed for reducing the pulp both tedious and uncertain, I abandoned it for a surer plan. The interior of the cavity is examined with a magnifying reflector, to keep in view the protruding pulp, then carefully remove as much of the decayed matter as possible without wounding the pulp. The acetate of morphia is now applied, and a suitable plug of cotton wool or scraped lint saturated in dissolved mastic, introduced over the powder to prevent its being taken up by the saliva. This must be renewed at intervals for two or three days; about that time the pulp will have receded, and left the

* A French instrument for cauterizing is described in the "Lancet"—No. March 28th, 1835.

cavity in a fit state to receive the stopping. But although the nerve is not present in the carious cavity, it would be imprudent to attempt to stop the tooth in the usual way, nor would gold be at all admissible, even with a lead floor. Nor are we bound to adopt that plan, having more manageable materials at hand in the amalgams; but also with these, the opening through which the pulp protruded, must be protected from the action of the stopping; and the system I adopt is to select a pellet of shot, which after flattening to the necessary thickness, then, with the stopping burnisher, I form it into a little cup. This cup is to be placed with its hollow over the pulp, similar to a dome. At first I found considerable difficulty in carrying this into execution. To place it on the exact spot was difficult, and, when there, to retain it *in situ.*—After trying different sorts of tongs without succeeding to my wish, I hit upon a plan so simple that the wonder was that I should have ever thought of any other. With the assistance of one of our stopping instruments,—a rectangle for the lower, an obtuse one for the other,—the point of which I touch with Canada Balsam, and apply it to the round top of the leaden cup, then carefully place it *in situ* over the opening at the bottom of the carious cavity, and hold it there. I then introduce the soft stopping, which I take care to press in gently, and carefully withdraw the instrument with which I have been holding the leaden cup, it being now securely fixed in its place. It may be as well to remark that the oxide should not be taken off the lead, as it prevents the quicksilver from amalgamating with it; and I am not certain that it has not some salutary influence upon the pulp. Mr. Koecker surmises that the acetate is formed while in the tooth. I doubt this, but it may be that the minute portion of arsenic that is introduced in the manufacture of shot may have some sedative influence upon the vessels of the tooth. It is necessary to observe that no pus is being formed in the cavity, which may be ascertained by a careful examination of the cotton when removed, as the stopping would not be successful even with the hollow cup, which would soon become filled, and cause great disturbance to the pulp.

A question will naturally arise, and with it a hope that the pulp thus protected would at once emulate the snail, and set about repairing its house, but I have never ascertained that this desirable result takes place, not having had an opportunity of examining any tooth afterwards, that had been so treated; but although I can only surmise, as regards the ossific deposit, I have personal experience of the success of the operation.—The anterior molar of my upper jaw was thus treated seven or eight years back, and has since continued perfectly free from uneasiness, and as useful in mastication as any sound tooth.

When the whole roof of the cavitas pulpæ has been removed by caries, the pulp frequently becomes tumid, filling nearly the whole carious hollow of the tooth; and although teeth in this state are entirely useless, yet, from the circumstance of their freedom from pain, and the usual dislike to extraction, we are often called upon to make some arrangement to prevent the food intruding into the cavity. The plan I have recommended could not be adopted in this case; so recourse must be had to get a gold cap, nicely fitted to spring upon the neck of the tooth, so to retain it firmly there during mastication. Caps are, no doubt, certain destruction to any tooth they may be applied to, unless removed and cleansed three or four times a day, to clear them of the acrid matter of which they generally are the depository. This acts upon the enamel of the tooth, and before long it is found entirely wasted away; but in the case now before us, this destructive agency would sometimes be hailed as a benefit, by ridding the mouth of a useless incumbrance, without pain to the patient.

21A, Saville-row, Regent-st.

